# Effects of Honokiol on CYP450 Activity and Transporter mRNA Expression in Type 2 Diabetic Rats

**DOI:** 10.3390/ijms19030815

**Published:** 2018-03-12

**Authors:** Junjun Wang, Ting Zhai, Yong Chen

**Affiliations:** Hubei Province Key Laboratory of Biotechnology of Chinese Traditional Medicine; Hubei Collaborative Innovation Center for Green Transformation of Bio-Resources, Hubei University, Wuhan 430062, China; wj-queen@163.com (J.W.); zhaitingjsw@126.com (T.Z.)

**Keywords:** honokiol, type 2 diabetic rats, CYP450, transporter

## Abstract

This study was aimed to clarify the effect of honokiol (Hon) on the activity of Cytochrome P450 (CYP450) enzymes, and the level of mRNA expression of liver and kidney transporters in type 2 diabetic rats induced by high-fat diet and strepotozotocin. Rats were randomly divided into normal control (NC) group, diabetic control (DC) group and Hon groups (*n* = 6). The activities of hepatic CYP1A2, CYP2E1, CYP2C, CYP2B, CYP3A and CYP4A, and the mRNA expression levels of hepatic and renal transporters, were determined. Compared to the NC group, the activities of CYP1A2, CYP2E1, CYP4A and CYP2C in DC group were increased by 2.36-, 2.10-, 2.55- and 1.86-fold, respectively. The mRNA expression levels of hepatic Oat2, Oatp2b1 and Oatp1a5, and renal Oct1, Octn2, Oatp2b1 and Oatp1a5, were significantly down-regulated, while the mRNA expression levels of hepatic Octn2, Oatp3a1, Oatp1a1 and Mdr2, and renal Oat2, Mrp4 and Bcrp, were significantly upregulated. Compared to the DC group, Hon treatment significantly inhibited the activity of hepatic CYP2E1, CYP4A, 3A and CYP1A2 by 45.6%, 29.2%, 22.7% and 20.7% in Hon high dose group, respectively. Moreover, Hon treatment significantly inhibited the mRNA expression levels of renal Bcrp and Mrp4 by 2.63-fold and 1.54-fold, while significantly upregulated the mRNA expression levels of hepatic Oat2 and Oatp2b1 by 1.52-fold and 1.54-fold in Hon high dose group, respectively. The results suggested that under the diabetes condition, the changes of CYP450 activity and transporter expression inevitably interfere the normal transport, metabolism and efficacy of drugs. The present work firstly reported that Hon treatment ameliorated the abnormal change of hepatic CYP activity (including CYP2E1, CYP4A and CYP1A2) and the transporter mRNA expression (including hepatic Oat2 and Oatp2b1, renal Bcrp and Mrp4) in type 2 diabetic rats induced by high-fat diet and strepotozotocin, which are associated with the occurrence and development of diabetes.

## 1. Introduction

With the improvement of living conditions, changes of diet structure and life style as well as the aggravation of aging, the incidence of diabetes increases rapidly in global, which makes diabetes become the third chronic disease severely threatened people’s health after tumor and cardiovascular disease. Type 2 diabetes mellitus (T2DM) is a disease with glucose and lipid metabolism disorder caused by the decrease of insulin sensitivity or β cells dysfunction, accounting for about 90% of all the patients with diabetes [1].

Cytochrome P450s (CYP450s) is one of the most important phase-I drug-metabolizing enzyme family that participates in the metabolism of most clinical drugs in liver and intestine. The transport protein is a large class of membrane proteins that mediate the exchange of chemical substances and signal inside and outside the biofilm. And they are the most important factors that influence the pharmacokinetic process of drugs. Besides exogenous compounds, species, age and sex, the expression and activity of CYP450 and transporters are also closely related with pathological state [2,3,4]. Studies showed that the protein expression level and enzyme activity of CYP3A in T2DM rats induced by high-fat diet and streptozotocin were upregulated [5]. The activities of hepatic CYP2E1, CYP1A2 and CYP3A were up-regulated [6,7], whereas the CYP2C activity was downregulated in T1DM rats induced by alloxan and streptozotocin [8,9]. The mRNA expression level of hepatic CYP 1a2 was up-regulated in New Zealand Obesity mice [10]. The hepatic CYP2c and 3a expression, and CYP3a activity were upregulated, while the CYP1a and 2e expression were downregulated in TSOD rats (obesity rats with T2DM) [11]. In addition, compared with db/db mice at the age of 10 weeks, the activity and expression of hepatic CYP2b10 and CYP2c9 at the age of 25 weeks decreased significantly while CYP4a10 activity increased obviously with the increase of age [12]. In db/db mice, the mRNA expression levels of hepatic and renal Oatp1a1, as well as that of hepatic Oatp1a4 were down-regulated [13]. In ob/ob mice, the mRNA and protein expression levels of hepatic Oatp1a1 as well as renal Oatp1a1mRNA expression levels were also down-regulated, and the expression of breast cancer resistance protein (Bcrp) and Oat2 in kidney of rats with T2DM were upregulated [14]. The above reports indicated that diabetes could cause the changes of CYP activity and transporter expression, and these will inevitably affect the normal transport, metabolism and effect of drugs. Therefore, the risk assessment of metabolic interaction in combined medication is of great importance, especially for the patients with diabetic complications.

Honokiol (Hon) is one of the primary active components of traditional Chinese medicine *Mangnolia officinalis*, which has extensive pharmacological effect like anti-inflammation [15], anti-tumor [16], anti-oxidation [17] and inducing cell apoptosis [18]. Our previous work indicated that Hon could significantly reduce the blood glucose and lipid level, improve glucose tolerance and inhibit hepatic oxidative stress injury of T2DM rats [19] which suggested that it has the effect of anti-diabetes. In addition, Hon could strongly inhibit the activity of CYP1A2, CYP2C8, CYP2C9 andCYP2C19 and moderately inhibited the activity of CYP2B6 in human microsomes [20]. Our previous study [21] also found that Hon has the inhibitory effect on the activity of CYP1A2 and 2E1 in normal rats. However, the effect of Hon on the activity of CYP450 in liver and the expression of transporters in liver and kidney of diabetic rats, and the correlation of this kind of influence with the anti-diabetic and anti-oxidative effects were still unknown so far. Therefore, the T2DM rats induced by high fatty diet and streptozotocin were taken as models in the present work and the effect of intragastric administration of Hon on the activities of CYP450 enzymes and expression of transporters in liver and kidney were studied, which could provide helpful information for guaranteeing the safety of clinical medication of patients with diabetes.

## 2. Results

### 2.1. Method Validation

Specificity of the method was evaluated by analyzing the mass chromatograms of blank liver microsomes, liver microsomes spiked with metabolite of probe substrate and liver microsomes sample after incubation with each probe substrate. No obvious interference peak was found near the peaks of tested analyte and IS (Figures S1–S6). The calibration curve, linear ranges, accuracy and precision of the 6 metabolites were also shown in Supplementary Materials (Table S1), and the results of the method validation could all meet the requirements of the test.

### 2.2. Effect of Hon on Hepatic CYP Activity

The effect of Hon on the activities of CYP1A2, 2E1, 2B, 3A, 2C and 4A were shown in Table 1. Compared to NC group, no obvious change for the activities of CYP2B and 3A, and the significantly increased activities of CYP1A2 (2.36-fold), 2E1 (2.10-fold), 4A (2.55-fold) and 2C (1.86-fold) were observed in DC group. Compared to DC group, Hon treatment group showed no obvious effect on CYP2B and CYP2C activity, but showed the inhibition effect on the activities of CYP2E1, 4A, 3A and 1A2 with the maximum inhibition rate of 45.6%, 29.2%, 22.7% and 20.7%, respectively.

### 2.3. Effect of T2DM on the mRNA Expression of Related Hepatic and Renal Transporters

It was reported that the protein expression of hepatic Mrp5 was decreased while the protein expression of renal Mrp4 and Bcrp were increased in T2DM rats [14]. The mRNA expression of Mrp4 was significantly up-regulated in kidney glomeruli of streptozotocin-induced diabetic rats [22]. Thus, we firstly studied the effect of T2DM on the mRNA expression of related hepatic and renal transporters in the present work, and the results were shown in Table 2. In DC group, the mRNA expression of Oat2, Oatp2b1 and Oatp1a5 were significantly down-regulated, while the Octn2, Otp3a1, Oato1a1 and Mdr2 mRNA expression was significantly increased in liver. Additionally, the Oat2, Mdr2 and Ntcp mRNA expression was significantly increased, whereas the mRNA expression of Oct1, Octn2, Oatp2b1 and Oatp1a5 were significantly decreased in kidney.

### 2.4. Effect of Hon on the mRNA Expression of Hepatic and Renal Transporters

According to the above experimental results, we chose some liver and kidney transporters (shown in Table 3 and Table 4) which were significant changed in the state of diabetes and further studied on the effects of Hon on the mRNA expression level of them. The effects of Hon on the mRNA expression of tested transporters were shown in Table 3 and Table 4. Compared to DC group, Hon could dose-dependently up-regulate the mRNA expression of hepatic Oat2 and Oatp2b1 almost to the normal level in the high dose group, no obvious effect was observed for the mRNA expression of other hepatic transporters (Table 3). However, Hon could down-regulate the mRNA expression of renal Bcrp (100 mg/kg) and Mrp4 (50 mg/kg) almost to the normal level, no obvious effect was observed for the mRNA expression of other renal transporters (Table 4).

## 3. Discussion

CYP enzymes participate in the metabolism of most clinical drugs. Our present work found that except the activities of hepatic CYP2B and 3A, the activities of hepatic CYP1A2, 2E1, 2C and 4A in the DC rats were all increased significantly as compared with those in the NC rats, suggesting that in the state of diabetes, the metabolic clearance of liver on the substrate drugs of CYP1A2, 2E1, 3C and 4A should be increased, which might affect the therapeutic action of drugs. After 8-week administration of Hon, the activity of hepatic CYP1A2, 2E1, 2C and 4A in the DC rats were dose-dependently inhibited while no obvious effect was observed for the hepatic CYP2B activity and only a significant inhibition for the hepatic CYP3A activity in the high dose of Hon.

CYP3A4 and 1A2 mediate the metabolism of estradiol and retinol in vivo. The serum levels of estradiol [23,24] and retinol [25,26,27] in the patients with diabetes were significantly lower than those in healthy people, suggesting that treatment of Hon might improve the decreased levels of estrogen and retinol caused by diabetes.

Human CYP2C family accounted for 20% of the total hepatic CYP content and participated in about 16% of the metabolism of clinical drugs. Among this family, CYP2C9 participated in the metabolism of various drugs for the treatment of diabetes (like glibenclamide and metformin) [28]. The rat CYP2C11 was highly similar to human CYP2C9 with the protein homology more than 77% [29]. Arachidonic acid, an endogenous substance, could be metabolized to EETs, Di-HETEs and 20-HETE by CYP4A, CYP2C and CYP2J in liver and kidney [30,31,32], and the metabolites were closely related with the occurrence and development of diabetes and its complications. For instance, 20-HETE plays an important role in the regulation of renal, cerebral and mesenteric arteries and coronary arteries and the increase of 20-HETE could induce the dysfunction of endothelial cells and lead to the incidence of diabetic hypertension at last [33]. The inhibition effect of Hon on the activity of CYP2C and 4A of rats with T2DM suggested that Hon played a positive role in the improvement of diabetic hypertension.

The activity of CYP2E1 and 4A in the liver microsomes could be induced in diabetic state [34,35,36]. The up-regulated expression of CYP2E1 and 4A was the important reason leading to lipid peroxidation damage of liver, insulin resistance and steatosis [37,38,39,40]. Our study found that Hon significantly inhibited the activity of CYP2E1 and CYP4A of rats with T2DM, which might be one of the most important mechanisms that Hon decreased the oxidative stress injury and insulin resistance in diabetic rats. In addition, CYP1A2 and CYP2E1 were involved in the metabolic activation of various cancer ogenic substances and protoxins [41,42]. Hon inhibited the activity of CYP1A2 and CYP2E1 of diabetic rats dosage-dependently indicating that Hon could inhibit the activation of some pre-cancerogenic and toxic substances induced by diabetes, which had protective effect on body.

To find new targets for the treatment of diabetes, the transporters that have not been reported to be associated with the incidence of diabetes were selected for the study of the effect of diabetes on the mRNA expression. And then we chose the transporters whose level of mRNA expression changed more than 2 folds in diabetic condition to study the effect of Hon on the mRNA expression of them. We studied the effect of Hon on mRNA expression of Octn2, Oatp2b1, Oatp1a5, Mrp4, Mrp5 and Bcrp in liver and kidney of rats with T2DM. The results showed that Hon with high dosage could reduce the effect of diabetes on the mRNA expression of Bcrp in kidney significantly. It was speculated that Hon might ameliorate disease condition of diabetes by regulating the expression level of some transporters. However, T2DM was often accompanied with complications and multiple drugs might be used concurrently so that there was risk of drug-drug interaction. The result of our study suggested that if the patients were given Hon and other drugs transported by Bcrp such as anti-diabetic drug glibenclamide at the same time, the dosage of drugs should be adjusted to reduce the drug–drug interaction and improve drug efficacy.

## 4. Materials and Methods

### 4.1. Chemicals and Reagents

Hon (purity ≥ 99%) was obtained from TCAS Chem Tech Co., Ltd. (Wuhan, China); Strepotozotocin, hydroxybupropion and 12-hydroxy lauric acid were purchased from Sigma Chem Co., Ltd. (St. Louis, MO, USA). Bupropion and 6β-hydroxy testosterone were purchased from Cayman Chem Co., (Ann Arbor, MI, USA). Phenacetinwas obtained from Alfa Aesar Chem Co., Ltd. (Tianjin, China); Chlorzoxazone was purchased from National Institute for the Control of Pharmaceutical and Biological Products (Beijing, China). Tolbutamide and testosterone were purchased from Dr. Ehrenstorfer GmbH, (Augsburg, Germany). Lauric acid was purchased from Nu-Chek Prep, Inc. (Los Angeles, CA, USA). Acetaminophen was purchased from TCI (Tokyo, Japan). 6-hydroxychlorzoxazoneand 4-hydroxytolbutamidewere purchased from TRC (Toronto, ON, Canada). NADPH was purchased from Biosharp (Hefei, China). HPLC-grade methanol and acetonitrile were purchased from Tedia Co., Inc. (Fairfield, OH, USA). RT-PCR Kit and Trans Start Green qPCR Super Mix were purchased from Toyobo Co., Ltd. (Osaka, Japan). Real-time fluorescent quantitative PCR primers were purchased from Sunny Biotech. Co., Ltd. (Shanghai, China). All other chemicals and reagents used in the study were of analytical grade.

### 4.2. Type 2 Diabetic Rats Model

50 Male SD rats (150–180 g) were purchased from the provincial Disease Prevention and Control Center of Hubei and were housed in SPF animal room at temperature (22 ± 2 °C) and 12/12 h day/night cycle. All procedures were approved by Ethic Committee of Hubei University (the approval number is 201507-003, and the approval date is 10 July 2015), and complied with health guidelines for the care and use of laboratory animals. 6 rats were fed with regular diet as NC group, the other 44 rats were fed with high fat diet (composed of 10% lard oil, 10% white sugar, 5% yolk power, 1% cholesterol and 74% regular diet) to induce type 2 diabetes. After 6 weeks, the rats fed with high fat diet were injected with 30 mg·kg^−1^ strepotozotocin (dissolved in citrate buffer, pH = 4.4) intraperitoneally, the normal rats were injected with same volume of citrate buffer. One week after STZ injection, the rats with glucose level ≥ 16.7 mmol·L^−1^ were selected for the further study. Animals were kept on their respective diet till the end of the study.

### 4.3. Animals Grouping and Treatment Protocol 

We are planning a study of a continuous response variable from independent control and experimental subjects with 1 control per experimental subject. Based on previous study, we estimated the sample size, and about 5 rats are needed in each group. Therefore, 24 diabetic rats were randomly divided into DC group and Hon treatment groups (25, 50 and 100 mg/kg/day) with 6 rats in each group. The normal rats were set as NC group. Hon (suspended in in 0.5% CMC-Na) was intragastrically administered to Hon treatment rats, the appropriate volume of 0.5% CMC-Na solution was intragastrically administered to NC and DC rats. At the end of the 8-week administration, rats of each group were sacrificed by decapitation. Livers and kidneys were isolated quickly and rinsed with ice-cold saline, and then stored in liquid nitrogen until analysis. Part of each liver was used to prepare liver microsomes, kidney and the rest part of the liver were used to extract total RNA.

### 4.4. Assay for CYP450 Activity

Liver microsomes of each rat was prepared by ultracentrifugation [43]. Phenacetin (50 μM), chlorzoxazone (75 μM), bupropion (75 μM), tolbutamide (150 μM), testosterone (75 μM) and lauric (100 μM) were chosen as the substrates of CYP1A2, CYP 2E1, CYP 2B, CYP2C, CYP3A and CYP4A. The incubation mixtures were prepared in a total volume of 200 μL as follows [44]: Rat liver microsomes (0.4 mg/mL, 0.4 mg/mL, 0.5 mg/mL, 0.5 mg/mL, 0.3 mg/mL and 0.3 mg/mL for CYP1A2, CYP 2E1, CYP2B, CYP2C, CYP3A and CYP4A), 5 mM MgCl_2_, 100 mM potassium phosphate buffer and probe substrate. The reaction was initiated by adding 1 mM NADPH and then incubated for 20 min, 15 min, 20 min, 10 min, 20 min and 20 min at 37 °C for CYP1A2, CYP2E1, CYP2B, CYP2C, CYP3A and CYP4A. Incubations were terminated by cooling on ice and adding 400 μL methanol with internal standard (IS). The mixture was centrifuged at 12,000× *g* for 10 min, and then 50 μL of the supernatant was evaporated by freeze-drying. The residue was dissolved in 100 μL mobile phase (shown in Table 5), and the content of metabolites of probe substrates were determined by HPLC-MS/MS.

### 4.5. LC/MS/MS Analysis 

A HPLC system couple to triple quadruple mass spectrometer (LC-MS/MS 8040; Shimadzu Corp., Kyoto, Japan) was used for analysis of the CYP450 enzyme activity. A VP-ODS column (2.0 mm × 150 mm, 4.6 µm; Shimadzu Corp., Kyoto, Japan) and a Shim-pack column guard column (2 mm × 5 mm, 4.6 µm; Shimadzu Corp., Kyoto, Japan) were used for LC separation. The gradient programs were shown in Table 5. The flow rate was 0.2 mL/min and the column temperature was 40 °C. The ESI source settings for ionization of the metabolites were as follows: nebulizer gas, 3 L/min; drying gas, 15.0 L/min; interface, 4.5 kV; CID gas, 230 kPa; DL temperature, 250 °C; heat block temperature, 400 °C. Quantitative analysis was performed by multiple reactions monitoring (MRM) or selective ion scan (SIM) [43]. Detection mode and MS transitions for the metabolites and internal standards were also shown in Table 5.

### 4.6. Assay for mRNA Expression of Transporters

100 mg cryopreserved liver or kidney tissue of each rat was taken to extract the total RNA according to the instruction of the Trizol kit (Invitrogen, CA, USA). The integrity of RNA was detected by 0.8% agarose gel electrophoresis and the purification of RNA was determined by nucleic acid protein analyzer. Fifteen µg total RNA was taken and reverse transcripted to cDNA after dilution according to the reverse transcription kit.

Primer 5.0 analysis software was used to design the primer sequence of reference gene Gapdh and each target gene (Table 6). The experimental condition of real-time quantitative PCR was: 95 °C, 5 min; 94 °C, 15 s; Tm, 30 s; 72 °C, 30 s, 40 cycles. The reaction system was 20 µL.

### 4.7. Data Analysis

All data were statistically analyzed by two-tailed unpaired *t*-test using SPSS 17.0 and are presented as the means ± standard errors.

## Figures and Tables

**Table 1:** Effects of Hon on activity of CYP450 of diabetic rats (*n* = 6).

Group	Enzyme Activity (ng/min/mg Protein)					
	CYP1A2	CYP2B	CYP2E1	CYP3A	CYP2C	CYP4A
NC	40.9 ± 4.8	6.96 ± 0.54	132.7 ± 33.1	3789.6 ± 733.5	59.9 ± 11.5	458.0 ± 50.3
DC	96.6 ± 12.6 **	7.20 ± 1.27	278.0 ± 48.6 **	3760.8 ± 588.1	111.5 ± 27.0 *	1167.4 ± 109.7 **
Hon25	84.0 ± 18.6 ^#^	7.46 ± 1.29	248.8 ± 28.5	3979.7 ± 263.9	102.2 ± 19.9	1170.7 ± 221.2
Hon50	82.0 ± 9.7 ^#^	7.25 ± 1.96	195.5 ± 50.8 ^#^	3324.2 ± 1116.1	99.2 ± 10.4	1091.4 ± 82.7
Hon100	74.7 ± 16.6 ^##^	7.13 ± 0.97	151.2 ± 45.1 ^##^	2983.8 ± 959.6 ^#^	98.2 ± 10.2	929.3 ± 262.7 ^##^

Hon 25, 50 and 100: diabetic rats treated with 25, 50 and 100 mg/kg of Hon, respectively. * *p* < 0. 05, ** *p* < 0.01 vs. NC; ^#^ *p* < 0.05, ^##^ *p* < 0.01 vs. DC.

**Table 2:** The mRNA expression of transporters in liver and kidney in diabetic rats (*n* = 6).

Gene	Fold Response			
	Liver		Kidney	
	NC	DC	NC	DC
*Oat2*	1.00 ± 0.32	0.64 ± 0.14 **	1.00 ± 0.25	1.56 ± 0.48 *
*Oct1*	1.00 ± 0.67	0.93 ± 0.27	1.00 ± 0.33	0.79 ± 0.14 *
*Octn1*	1.00 ± 0.46	1.60 ± 0.71	1.00 ± 0.30	1.13 ± 0.49
*Octn2*	1.00 ± 0.67	3.24 ± 0.44 *	1.00 ± 0.17	0.54 ± 0.12 **
*Oatp2b1*	1.00 ± 0.45	0.64 ± 0.21 **	1.00 ± 0.14	0.78 ± 0.16 *
*Oatp1a5*	1.00 ± 0.46	0.47 ± 0.14 *	1.00 ± 0.56	0.50 ± 0.16 *
*Oatp3a1*	1.00 ± 0.78	1.65 ± 0.15 *	1.00 ± 0.34	1.27 ± 0.63
*Oatp1a1*	1.00 ± 0.64	1.34 ± 0.06 *	1.00 ± 0.47	0.85 ± 0.58
*Oatp1a4*	1.00 ± 0.34	1.43 ± 0.18	1.00 ± 0.73	0.62 ± 0.43
*Mdr2*	1.00 ± 0.62	1.81 ± 0.15 *	1.00 ± 0.33	1.38 ± 0.22 *
*Ntcp*	1.00 ± 0.45	1.31 ± 0.69	1.00 ± 0.63	1.76 ± 0.22 *
*Mate1*	/	/	1.00 ± 0.32	0.87 ± 0.48

/: not applicable; * *p* < 0.05, ** *p* < 0.01 vs. NC.

**Table 3:** Effect of Hon on the mRNA expression of hepatic transporters in T2DM rats (*n* = 6).

Gene	Fold Response				
	NC	DC	Hon25	Hon50	Hon100
*Oat2*	1.00 ± 0.11	0.57 ± 0.24 **	0.61 ± 0.68	0.61 ± 0.47	0.87 ± 0.38 ^#^
*Octn2*	1.00 ± 0.56	2.71 ± 1.04 *	2.36 ± 0.32	2.06 ± 0.31	2.82 ± 0.36
*Oatp2b1*	1.00 ± 0.27	0.61 ± 0.41 **	0.71 ± 0.85	0.74 ± 0.35	0.94 ± 0.41 ^#^
*Oatp1a5*	1.00 ± 0.05	0.33 ± 0.29 *	0.22 ± 0.41	0.17 ± 0.45	0.10 ± 0.28
*Mrp5*	1.00 ± 0.44	1.26 ± 0.43	0.94 ± 0.16	1.09 ± 0.60	1.01 ± 0.60

* *p* < 0.05, ** *p* < 0.01 vs. NC; ^#^ *p* < 0.05.

**Table 4:** Effect of Hon on the mRNA expression of renal transporters in T2DM rats (*n* = 6).

Gene	Fold Response				
	NC	DC	Hon25	Hon50	Hon100
*Oat2*	1.00 ± 0.53	2.70 ± 0.52 **	2.43 ± 0.96	2.62 ± 0.82	2.71 ± 0.69
*Octn2*	1.00 ± 0.55	0.59 ± 0.46 *	0.50 ± 0.37	0.59 ± 0.49	0.57 ± 0.29
*Oatp2b1*	1.00 ± 0.33	0.70 ± 0.46 *	0.77 ± 0.34	0.84 ± 0.58	0.71 ± 0.16
*Oatp1a5*	1.00 ± 0.21	0.61 ± 0.15 *	0.57 ± 0.41	0.94 ± 0.16	0.26 ± 0.20
*Mrp4*	1.00 ± 0.55	1.73 ± 0.56 *	1.37 ± 0.59	1.12 ± 0.73 ^#^	1.22 ± 0.23
*Bcrp*	1.00 ± 0.21	2.23 ± 0.81 **	1.69 ± 0.59	0.85 ± 0.22	0.92 ± 0.14 ^#^

* *p* < 0.05, ** *p* < 0.01 vs. NC; ^#^ *p* < 0.05.

**Table 5:** The methods of the content analysis of metabolites tested.

Metabolite/IS	Mobile Phase	Flow Rate (mL/min)	Detection
acetaminophen/ 6β-hydroxytestosterone	A: 0. 1% formic acid in water; B: acetonitrile (0–5 min, 15–85% B; 5–6 min, 85% B; 6–6.01 min, 15% B; 6–11 min, 15% B)	0.2	MRM+: 152. 0→110. 1/305. 0→269. 0
6-hydroxychlorzoxazone/ 4′-hydroxytolbutamide	A: 0. 1% formic acid in water; B: acetonitrile (0–5 min, 20–70% B; 5–6 min, 70% B; 6–6.01 min, 20% B; 6–11min, 20% B)	0.2	MRM−: 184. 0→120. 1/285. 0→186. 1
hydroxybupropion/acetaminophen	A: 0. 1% formic acid in water; B: acetonitrile (0–5 min, 15–85% B; 5–6 min, 85% B; 6–6.01 min, 15% B; 6–11 min, 15% B)	0.2	MRM+: 256. 0→238. 0/152. 0→110. 1
6β-hydroxytestosterone/ acetaminophen	A: 0. 1% formic acid in water; B: acetonitrile (0–5 min, 15–85% B; 5–6 min, 85% B; 6–6.01 min, 15% B; 6–11 min, 15% B)	0.2	MRM+: 305. 0→269. 0/152. 0→110. 1
4′-hydroxytolbutamide/6-hydroxychlorzoxazone	A: 0. 1% formic acid in water; B: acetonitrile (0–5 min, 20–70% B; 5–6 min, 70% B; 6–6.01 min, 20% B; 6–11 min, 20% B)	0.2	MRM−: 285. 0→186. 1/184. 0→120. 1
12-hydroxylauric acid/ 6-hydroxychiorzoxazone	A water; B: methanol (0–4 min, 40–60% B; 4–5 min, 60–90% B; 5–9 min, 90% B; 9–9.01 min, 40% B; 9–14 min, 40% B)	0.2	SIM−: 215. 0/184. 0

**Table 6:** Primer sequences used for real-time quantitative PCR.

Gene	Forward Primer 5′ to 3′	Reverse Primer 5′ to 3′
*Oat2*	GCTGCATGATGGTGTGGTTT	CGGCGCACAAGGAAGTAGAC
*Oct1*	TGGCCGTAAGCTCTGTCTCT	TCAAGGTATAGCCGGACACC
*Octn1*	TGATGTCTGTGATGCTGTGG	ATATATCTCCGACGCAGGGTTC
*Octn2*	AACAATGGCAAATCCAAAGC	CATCCGTGAGCATGTGAGAC
*Oatp2b1*	CCGCTACGACCACAGCA	CCAAGACCTTCTGCCTGA
*Oatp1a5*	CGCTTTGATAGACAGAACCT	AGTAGCAGCATGAAACGACA
*Oatp3a1*	TTCTGCTCCTTCGTTTGTT	GGTTTTTGATGTAGCGTTT
*Oatp1a1*	TCAACCAAAGCACAAAGCAG	CCTAGGCATAGGCATTTGGA
*Oatp1a4*	ATGGCCTGGCATACATGTCA	GGGAACTGGAATGTCCTCGTA
*Mdr2*	ACTGTCCGGAATGCAGATGTC	TCTTTATCAGCTCACTGTGGCTT
*Ntcp*	ATGCCCTTCTCTGGCTTTCT	GCTCCATGGTTCTGATGGTT
*Mate1*	CTCTTCATCAACACCGAGCA	ACCCATCACCCCAAGATGTA
*Mrp4*	AATGGACACTGAACTAGCAGAATCTG	CCCGGATTTTCTGTTGTATTAACTC
*Mrp5*	CCACCATCCATGCCTATAACAA	CCCCGTGGTGGTGATCAG
*Bcrp*	CAGCAGGTTACCACTGTGAG	TTCCCCTCTGTTTAACATTACA
*Gapdh*	ATGGGAAGCTGGTCATCAAC	GTGGTTCACACCCATCACAA

## Supplementary Materials

Supplementary materials can be found at http://www.mdpi.com/1422-0067/19/3/815/s1.
