# Supplementary material for: Effects of Honokiol on CYP450 Activity and Transporter mRNA Expression in Type 2 Diabetic Rats
Source: Int J Mol Sci. 2018 Mar 12;19(3):815. doi: 10.3390/ijms19030815 (PMC5877676; doi:10.3390/ijms19030815)
Supplement: Supplementary file 1 [file ijms-19-00815-s001.pdf]

1. Specificity

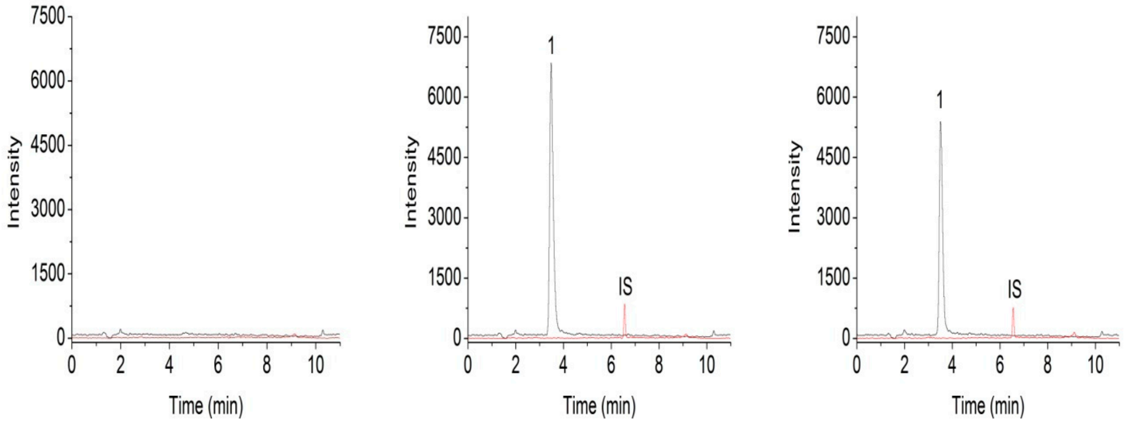

A B C

Figure S1. Typical chromatograms of acetaminophen in rat microsomes. A: blank microsomes; B: blank microsomes spiked with acetaminophen and IS(6β-hydroxytestosterone); C: microsomes incubated with phenacetin.

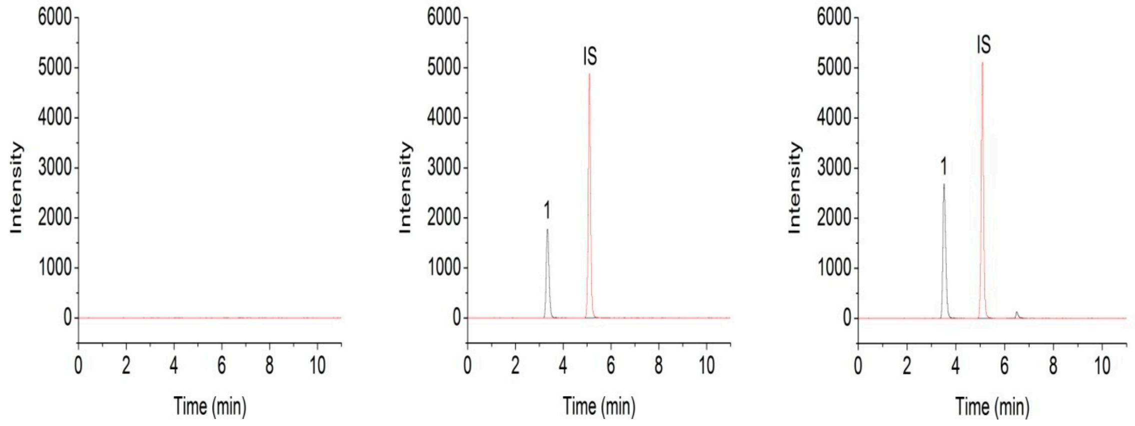

A B C

Figure S2. Typical chromatograms of 6-hydroxychlorzoxazone in rat microsomes. A: blank microsomes; B: blank microsomes spiked with 6-hydroxychlorzoxazone and IS(4′-hydroxytolbutamide); C: microsomes incubated with chlorzoxazone.

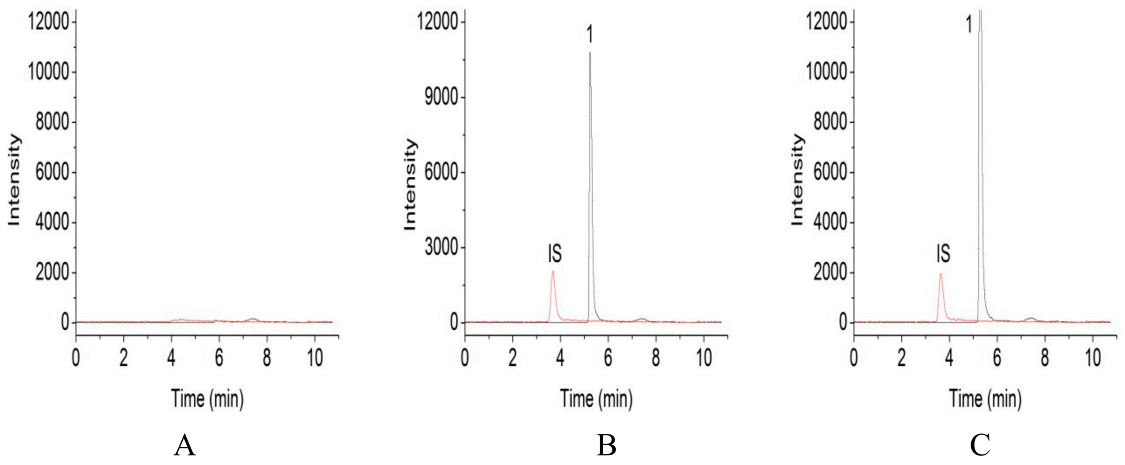

Figure S3. Typical chromatograms of hydroxybupropion in rat microsomes. A: blank microsomes; B: blank microsomes spiked with hydroxybupropion and IS(acetaminophen); C: microsomes incubated with bupropion.

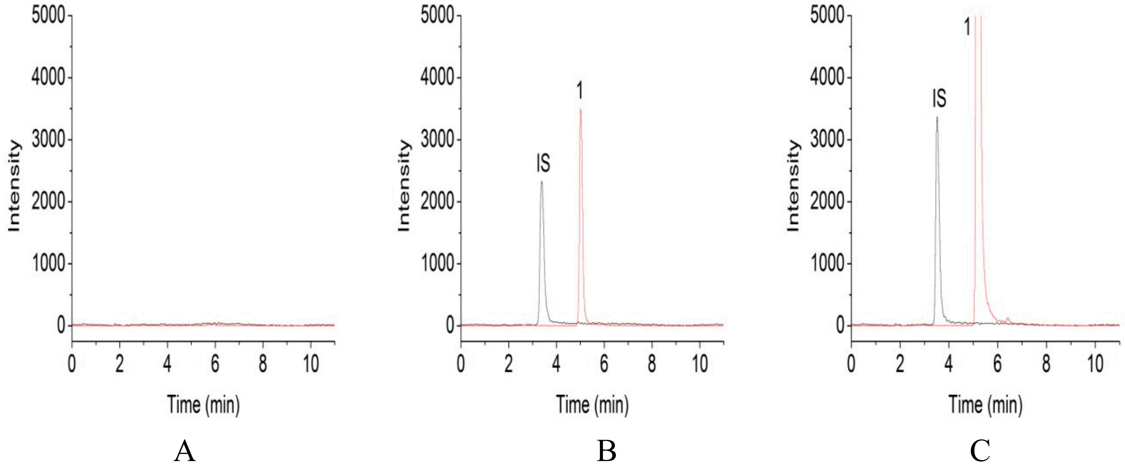

Figure S4. Typical chromatograms of 4′-hydroxytolbutamide in rat microsomes. A: blank microsomes; B: blank microsomes spiked with 4′-hydroxytolbutamide and IS (6-hydroxychlorzoxazone); C: microsomes incubated with tolbutamide.

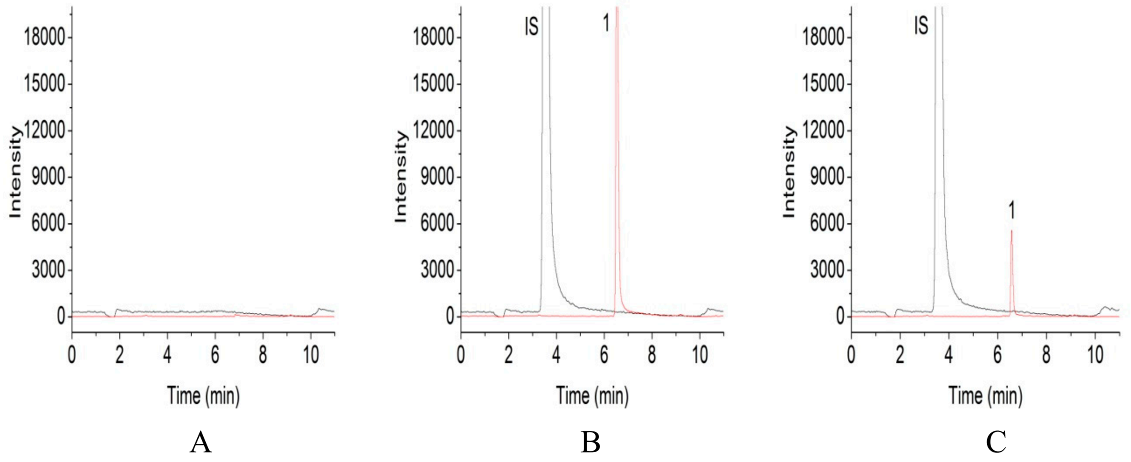

Figure S5. Typical chromatograms of 6β-hydroxytestosterone in rat microsomes. A: blank microsomes; B: blank microsomes spiked with acetaminophen and IS(acetaminophen); C: microsomes incubated with testosterone.

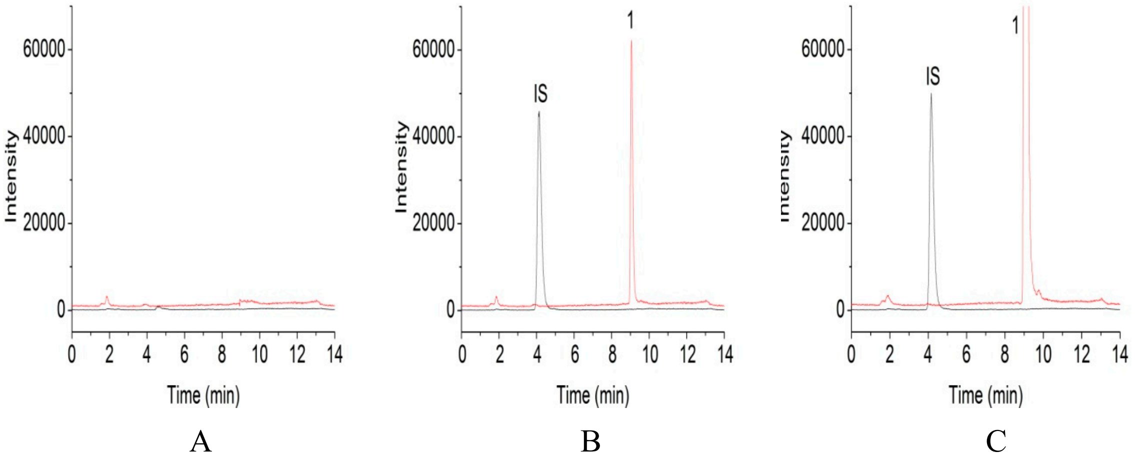

Figure S6. Typical chromatograms of 12-hydroxylauric acid in rat microsomes. A: blank microsomes; B: blank microsomes spiked with acetaminophen and IS (6-hydroxychlorzoxazone); C: microsomes incubated with lauric acid.

2. Calibration curves, accuracy and the precision

The linear ranges of the 6 metabolites mentioned in the manuscript were 0.16 μM-10 μM, 0.47 μM-30 μM, 0.94 μM-30 μM, 0.19 μM-2 μM, 0.10 μM-3.20 μM and 0.78 μM-50 μM, respectively. The linear equation of calibration curves, accuracy and the precision of the metabolites were presented in Table S1.

Table S1. Calibration curves, accuracy and precision for the tested metabolites (n=6),.

Metabolite	Calibration Curves	QC/μM	Intra-Assay/%		Inter-Assay/%	
			RSD	Accurary	RSD	Accurary
Acetaminophen	*Y=131.3x+53.7* r^2=1.000	0.32	11.1	95.6±10.6	3.2	103.2±3.3
		1.25	6.3	99.0±6.2	2.7	96.4±2.6
		10.0	7.7	99.6±7.7	5.9	105.1±6.2
6-hydroxychlorzoxazone	*Y=1561.0x+11.5* r^2=0.999	0.94	6.9	96.6±6.7	3.9	97.8±3.8
		3.75	2.7	97.6±2.6	2.3	98.6±2.3
		30.0	4.6	99.3±4.6	1.8	96.2±1.7
4′-hydroxytolbutamide	*Y=110.8x-65.2* r^2=0.996	0.38	13.9	112.8±5.7	6.1	102.3±6.2
		1.50	6.5	102.1±6.6	5.4	99.6±5.4
		12.00	3.1	109.2±4.7	7.7	97.3±7.5
6β-hydroxytestosterone	*Y=469.5x-0.43* r^2=0.992	0.94	12.3	96.0±11.8	6.5	95.1±6.2
		3.75	11.5	101.2±11.6	5.3	99.8±5.3
		30.00	7.0	104.3±7.3	4.2	103.2±4.3
Hydroxybupropion	*Y=18.29X-12.94* r^2= 0.999	0.20	6.8	91.4±6.3	5.1	93.6±4.8
		0.80	3.8	105.0±4.0	2.0	102.6±2.1
		3.20	2.3	98.1±2.2	1.4	103.6±1.4
12-hydroxylauric acid	*Y=1132.1X+7.32* r^2 = 0.996	1.56	5.7	113.2±6.4	2.0	100.1±2.0
		6.25	7.2	100.3±7.2	3.4	104.5±3.6
		50.0	3.0	91.4±2.7	3.7	103.8±3.8
